# Lipidomic studies revealing serological markers associated with the occurrence of retinopathy in type 2 diabetes

**DOI:** 10.1186/s12967-024-05274-9

**Published:** 2024-05-13

**Authors:** Mingqian He, Guixue Hou, Mengmeng Liu, Zhaoyi Peng, Hui Guo, Yue Wang, Jing Sui, Hui Liu, Xiaoming Yin, Meng Zhang, Ziyi Chen, Patrick C.N. Rensen, Liang Lin, Yanan Wang, Bingyin Shi

**Affiliations:** 1 Department of Endocrinology, the First Affiliated Hospital of Xi’an JiaoTong University, No.277, West Yanta Road, Xi’an, Shaanxi 710061 P.R. China; 2 BGI-SHENZHEN, No. 21 Hongan 3rd Street, Yantian District, Shenzhen, Guangdong 518083 P.R. China; 3 Department of Endocrinology and International Medical Center, the First Affiliated Hospital of Xi’an JiaoTong University, No.277, West Yanta Road, Xi’an, Shaanxi 710061 P.R. China; 4 Biobank, The First Affiliated Hospital of Xi’an JiaoTong University, Xi’an, Shaanxi 710061 China; 5 Chengdu HuiXin Life Technology, Chengdu, Sichuan 610091 P.R. China; 6 Department of Medicine, Division of Endocrinology, Leiden University Medical Center, P.O. Box 9600, Leiden, 2300 RA The Netherlands; 7 Med-X institute, Center for Immunological and Metabolic Diseases, the First Affiliated Hospital of Xi’an JiaoTong University, Xi’an JiaoTong university, Xi’an, Shaanxi 710061 P.R. China; 8 Building NO.7, BGI Park, No. 21 Hongan 3rd Street, Yantian District, Shenzhen, Guangdong 518083 P.R. China

**Keywords:** Type 2 diabetes mellitus, Retinopathy, Lipidomics, Serological markers, Ceramide

## Abstract

**Purpose:**

The duration of type 2 diabetes mellitus (T2DM) and blood glucose levels have a significant impact on the development of T2DM complications. However, currently known risk factors are not good predictors of the onset or progression of diabetic retinopathy (DR). Therefore, we aimed to investigate the differences in the serum lipid composition in patients with T2DM, without and with DR, and search for potential serological indicators associated with the development of DR.

**Methods:**

A total of 622 patients with T2DM hospitalized in the Department of Endocrinology of the First Affiliated Hospital of Xi’an JiaoTong University were selected as the discovery set. One-to-one case–control matching was performed according to the traditional risk factors for DR (i.e., age, duration of diabetes, HbA1c level, and hypertension). All cases with comorbid chronic kidney disease were excluded to eliminate confounding factors. A total of 42 pairs were successfully matched. T2DM patients with DR (DR group) were the case group, and T2DM patients without DR (NDR group) served as control subjects. Ultra-performance liquid chromatography–mass spectrometry (LC–MS/MS) was used for untargeted lipidomics analysis on serum, and a partial least squares discriminant analysis (PLS-DA) model was established to screen differential lipid molecules based on variable importance in the projection (VIP) > 1. An additional 531 T2DM patients were selected as the validation set. Next, 1:1 propensity score matching (PSM) was performed for the traditional risk factors for DR, and a combined 95 pairings in the NDR and DR groups were successfully matched. The screened differential lipid molecules were validated by multiple reaction monitoring (MRM) quantification based on mass spectrometry.

**Results:**

The discovery set showed no differences in traditional risk factors associated with the development of DR (i.e., age, disease duration, HbA1c, blood pressure, and glomerular filtration rate). In the DR group compared with the NDR group, the levels of three ceramides (Cer) and seven sphingomyelins (SM) were significantly lower, and one phosphatidylcholine (PC), two lysophosphatidylcholines (LPC), and two SMs were significantly higher. Furthermore, evaluation of these 15 differential lipid molecules in the validation sample set showed that three Cer and SM(d18:1/24:1) molecules were substantially lower in the DR group. After excluding other confounding factors (e.g., sex, BMI, lipid-lowering drug therapy, and lipid levels), multifactorial logistic regression analysis revealed that a lower abundance of two ceramides, i.e., Cer(d18:0/22:0) and Cer(d18:0/24:0), was an independent risk factor for the occurrence of DR in T2DM patients.

**Conclusion:**

Disturbances in lipid metabolism are closely associated with the occurrence of DR in patients with T2DM, especially in ceramides. Our study revealed for the first time that Cer(d18:0/22:0) and Cer(d18:0/24:0) might be potential serological markers for the diagnosis of DR occurrence in T2DM patients, providing new ideas for the early diagnosis of DR.

**Supplementary Information:**

The online version contains supplementary material available at 10.1186/s12967-024-05274-9.

## Introduction

Type 2 diabetes mellitus (T2DM) is a common chronic disease in many countries, and its prevalence is growing as people’s lifestyles are changing [1]. Diabetes causes various complications, classified as either macrovascular complications (such as cardiovascular disease and stroke) or microvascular complications (such as kidney disease) [2]. Diabetic retinopathy (DR), a specific microvascular complication of diabetes, is the most common cause of vision loss in people of working age [3, 4]. Poor glycemic control, hypertension, and diabetes duration are major risk factors for DR [5]. Although intensive risk factor control reduces the risk of DR progression and vision loss, many diabetic patients continue to develop DR with strict glycemic and blood pressure control [6]. Despite increasing research supporting the efficacy of routine DR screening to prevent DR and early treatment to reduce the risk of vision loss, there are no specific biomarkers for diagnosing the onset and early progression of DR. Additionally, new and more effective strategies are awaited to prevent and treat the progression of DR.

Accumulating evidence suggests that disruption in lipid metabolism is an early event in the pathogenesis of diabetes complications. Previous studies found that levels of multiple lipid species, including glycerophospholipids, sphingolipids and glycerolipids, are critical risk factors for T2DM and its complications [7, 8]. Lysophosphatidylcholine (LPC) is a main glycerophospholipid known for its essential role in lipid and glucose metabolism, and LPC has been intensively studied in the development of metabolic diseases including T2DM [9]. Sphingolipids, including ceramides (Cer), sphingomyelins (SM) and gangliosides, have a variety of intra- and extracellular effects on glucose homeostasis and metabolic disease [10] Numerous studies suggest Cer, a crucial lipid intermediate in sphingolipid metabolism, is a major contributing factor for insulin resistance, and inhibition or depletion of enzymes driving *de novo* ceramide synthesis can prevent the development of diabetes in mice [7, 11, 12]. In contrast, a decrease in very long chain Cer is correlated with the development of macroalbuminuria in diabetes [13]. Accelerated sphingolipid catabolism’ leading to an increase in glucosylceramide or glycosphingolipids might contribute to the neuronal pathologies of DR [14]. In addition, SM produced by the transfer of a phosphocholine moiety from phosphatidylcholine to the ceramide backbone has been linked to insulin resistance [15, 16] and is also an independent marker of cardiovascular disease [17]. Thus, dysregulated lipid metabolism is a major contributor to the pathogenesis of T2DM and its complications, and specific lipid species that are responsible for the occurrence of DR are rather obscure.

Lipidomics offers solid platforms for identifying novel lipid mediates in biochemical processes of lipid metabolism, thus providing new opportunities for disease prediction and detection [18, 19]. Lipidome analysis is performed by liquid chromatography and electrospray ionization-tandem mass spectrometry (LC–MS/MS) for molecular lipid identification and quantification and multiple reaction monitoring (MRM) for targeted quantification of those lipid species. Lipid-based biomarkers offer unique options for precision medicine by providing sensitive diagnostic tools for disease prediction and monitoring [20]. Using a quantitative metabolomics approach, Emil et al. compared the aqueous humor and serum concentrations of metabolites in senior adults with an without diabetes who underwent cataract surgery [21]. However, the field of lipidomics studies of DR is still in its early stages, with few studies published and little replication of results [22].

In this study, we aimed to find reliable serum lipid-based biomarkers for the presence of DR in patients with T2DM by using two cohorts. To this end, serum samples of the discovery cohort was subjected to untargeted lipidomics analysis to search for differentially abundant lipids between individuals without and with DR. In the validation cohort, the observed differential lipid molecules were validated using mass spectrometry MRM targeting techniques. We hypothesized that DR has a distinctive serum lipid signature and that particular lipid species can act as biomarkers for T2DM patients with DR.

## Research design and methods

### Participants

A total of 622 participants with T2DM hospitalized in the Endocrinology Department of the First Affiliated Hospital of Xi’an JiaoTong University were screened as the discovery set. Participants with chronic kidney disease [estimated glomerular filtration rate (eGFR) < 90 (mL/min/1.73 m^2^)] were excluded from the selection. We conducted pair matching according to the traditional risk factors for DR (including age, duration of diabetes, HbA1c level, and hypertension). For the discovery cohort, we selected 42 T2DM patients with DR (DR group). The control participants were 42 T2DM patients without DR (NDR group), and they were matched to patients in the DR group by age (in 5-year bands), diabetes duration (in 5-year bands), HbA1c levels (in 0.5% bands), and hypertension status.

Lipid markers of DR identified from the discovery cohort were quantified in a separate sample cohort (validation cohort). We first screened 531 T2DM patients. Individuals with chronic kidney disease [eGFR < 90 (mL/min/1.73 m^2^)] were excluded from the selection. Then, we conducted 1:1 propensity score matching (PSM) (matching tolerance = 0.02) by age, diabetes duration, HbA1c level, hypertension status, sex, BMI, systolic blood pressure (SBP), diastolic blood pressure (DBP), and eGFR. For the validation cohort, 95 T2DM patients with DR (DR group) and 95 T2DM patients without DR (NDR group) were included.

### Sample collection

Fasting blood samples and clinical data were collected from the individuals. All blood samples were collected at the First Affiliated Hospital of Xi’an JiaoTong University physical examination center. Blood samples were centrifuged for 20 min at 1500 rpm and 4 °C. Then, serum was collected and stored at -80 °C until analysis. HbA1c was measured using an automatic HbA1c analyzer (TOSOH BIOSCIENCE, INC.; HLC-723G8). Total cholesterol (CHOL), triglyceride (TG), high density lipoprotein-cholesterol (HDL-c), low density lipoprotein-cholesterol (LDL-c), uric acid (UA), aspartate aminotransferase (AST), alanine aminotransferase (ALT), alkaline phosphatase (ALP), gamma-glutamyl transpeptidase (GGT), total bilirubin (TBIL), direct bilirubin (DBIL), total protein (TP), albumin (ALB), glucose (GLU), blood urea nitrogen (BUN), creatinine (CRE) were measured using standard reagents on an automatic biochemistry analyzer (HITACHI, Inc.; LAbOSPECT, 008AS). Blood pressure was measured in triplicate using an Omron HBP-9020 digital automatic blood pressure machine (Kyoto, Japan).

### Lipid extraction

The serum samples were thawed slowly at 4 °C, 100 µL of the sample was placed in a 96-well plate, 300 µL of isopropanol (prechilled at -20 °C) spiked with internal standards (SPLASH® LIPIDOMIX® Mass Spec Standard, Avanti, USA) was added, and the samples were vortexed and mixed for 1 min and then centrifuged at 4 °C for 20 min at 4000 rcf after resting overnight at -20 °C as previously reported [23]. The supernatant was injected for LC–MS/MS analysis, and 10 µL of each supernatant was mixed into quality control (QC) samples to assess the reproducibility and stability of the LC–MS analysis process.

### LC–MS/MS analysis

Lipids were separated and detected by an UPLC (CSH C18 column, 1.7 μm 2.1*100 mm, Waters, USA) equipped with a Q Exactive Plus high-resolution mass spectrometer (Thermo Fisher Scientific, USA) as previously reported [24]. The following gradient was used for elution: 0–2 min, 40-43% mobile phase B (10 mM ammonia formate, 0.1% formic acid, 90% isopropyl alcohol, and 10% acetonitrile); 2–2.1 min, 43-50% liquid B; 2.1–7 min, 50-54% solution B; 7–7.1 min, 54-70% liquid B; 7.1–13 min, 70-99% liquid B with a flow rate of 0.35 mL/min. Mobile phase A was an aqueous solution containing 10 mM ammonia formate, 0.1% formic acid and 60% acetonitrile in water.

All samples were analyzed in data-dependent acquisition (DDA) mode with the following positive/negative ionization settings: spray voltage, 3.8/–3.2 kV; aux gas heater temperature, 350 °C; and capillary temperature, 320 °C. The full scan mass range was 200–2000 m/z with 70,000 mass resolution at m/z 200 and AGC set to 3e6 with a maximum ion injection time of 100 ms. The top three precursors were selected for subsequent MS fragmentation with a maximum ion injection time of 50 ms and resolution of 17,500 at m/z 200, and the AGC was 1e5. The stepped normalized collision energy was set to 15, 30, and 45 eV.

### Data preprocessing and quality control

The raw data obtained from the LC–MS/MS detection were imported into LipidSearch v.4.1 (Thermo Fisher Scientific, USA) for lipid identification and quantification. The following parameters were used for lipid identification and peak extraction: the type of identification was Product, the mass deviation of the parent and daughter ions was 5 ppm, and the response threshold was set to 5.0% of the relative response deviation of the daughter ions; the quantitative parameters were set to calculate the peak areas of all identified lipids, and the peak extraction mass deviation was set to 5 ppm. For ESI + data, [M + H]+, [M + NH4]+, and [M + Na] + were selected as adducts, while for ESI- data, [M-H]-, [M-2 H]-, and [M-HCOO]- were selected as adducts. The peak alignment was performed for all identified lipids, and those not marked as “rejected” were considered for inclusion in the subsequent analysis.

For data preprocessing, raw data exported from LipidSearch were further analyzed by meta X [25]. The data preprocessing included (1) Removing lipid molecules with more than 50% missing information in QC samples and more than 80% missing information in experimental samples (i.e., LipidIon in the table); (2) Filling the missing values using the k-nearest neighbor (KNN) algorithm; (3) Correcting the batch effect using quality control-based robust LOESS signal correction (QC-RLSC); (4) Using probabilistic quotient normalization (PQN) to normalize the data to obtain the relative peak areas; and (5) Removing the lipid molecules with a coefficient of variation (CV) greater than 30% of the relative peak areas from all QC samples.

Data quality was assessed by the reproducibility of QC sample assays. The assessment included chromatogram overlap of QC samples, principal component analysis (PCA), number of extracted peaks, and differences in peak response intensity.

### Data processing

A combination of multivariate statistical analysis and univariate analysis was used to screen for lipids of which the abundance differed between groups. The multivariate statistical analysis methods used were principal component analysis (PCA) and partial least squares method-discriminant analysis (PLS-DA). PCA is an unsupervised pattern recognition method, and PLS-DA is a supervised pattern recognition method. The univariate analyses were fold change (FC) and Student’s t test. The FC was obtained by fold change analysis, and the *p* value pairs of the t test were corrected for the false discovery rate (FDR) to obtain a q-value. The differential lipid molecule screening conditions were as follows: (1) variable importance in the projection (VIP) ≥ 1 for the first two principal components of the PLS-DA model; (2) fold change ≥ 1.2 or ≤ 0.83; and (3) *p* value < 0.05.

### Targeted lipid quantification by MRM in validation samples

The identified differential lipids were further quantified by multiple reaction monitoring (MRM). For lipid extraction, the procedure was consistent with the untargeted experiment as described. The MRM transition list is shown in Table S1. For MRM quantification, all validation samples were analyzed on a QTRAP 5500 mass spectrometer with a CSH C18 column (1.7 μm 2.1*100 mm, Waters, USA) for separation. All lipids were subjected to targeted quantification in ESI + mode with a specific transition setting.

### Statistical analysis

The clinical data of samples are presented as the mean ± standard deviation (SD) for normally distributed variables or the median (interquartile range) for abnormal distribution. Comparisons between the case group and the control group were made using a two-tailed t test or Mann-Whitney U test for continuous data and the X^2^ test for categorical data. The calculation of the area under the curve (AUC) in receiver operating characteristic (ROC) curve analysis was used to evaluate the discriminatory ability of the markers. Logistic regression models were applied to assess the relationship between lipid molecules and the presence of DR. The odds ratios (ORs) with 95% confidence intervals (CIs) were calculated for the molecules with 1-SD changes. The known risk factors for DR, such as CHOL, TGs, LDL-c, and HDL-c, were added to multivariate logistic regression to calculate the adjusted odds ratios. Ordinal logistic regression models were used to assess the relationships between lipid molecules and DR stages [NDR, nonproliferative DR (NPDR) and proliferative DR (PDR)].

## Results

### Characteristics of the discovery cohort

Table 1 shows the clinical characteristics of individuals selected for the discovery cohort. There were no significant differences in age and sex between the DR and NDR groups. In fact, these groups were comparable for most metabolic characteristics, such as BMI, diabetes duration, and HbA1c, and there were no significant between-group differences for hypertension status, antihypertensive agent use, hypoglycemic therapy status or NSAID use. The blood pressure and glucose of the participants were treated and controlled. Compared with control subjects with T2DM, T2DM patients with DR had higher levels of LDL-c levels, AST, TBIL, and BUN (Table 1 and Table S2).

**Table 1:** Clinical characteristics of the discovery cohort

	NDR group	DR group	*P* value
Sex (male/total)	25/42	33/42	0.059
Age (years)	57.7 ± 10.0	56.5 ± 10.8	0.593
BMI (kg/m^2^)	25.4 ± 3.3	25.1 ± 3.2	0.697
Diabetes duration (years)	11.4 ± 6.7	12.2 ± 5.9	0.557
HbA1c (%)	8.1 ± 1.6	8.1 ± 1.6	0.945
Hypoglycemic therapy			0.086
Oral hypoglycemic agents	17	10	
Insulin therapy	6	3	
Combination therapy	19	29	
Hypertension status	28/42	27/42	0.818
Antihypertensive agents	24/42	27/42	0.503
Lipid-lowering agents			0.708
Statins	36	38	
Fibrates	2	1	
Others	1	0	
Use of NSAIDs	30/42	31/42	0.807
Systolic BP (mmHg)	136 ± 15	140 ± 24	0.370
Diastolic BP (mmHg)	77 ± 11	79 ± 10	0.869
eGFR (mL/min/1.73 m^2^)	129 ± 34	121 ± 35	0.334
CHOL (mmol/L)	3.87 ± 0.98	4.20 ± 1.24	0.176
TG (mmol/L)	1.47 (1.78)	1.65 (1.36)	0.960
HDL-c (mmol/L)	0.95 ± 0.22	0.97 ± 0.23	0.707
LDL-c (mmol/L)	2.20 ± 0.86	2.66 ± 1.12	**0.039**

NDR: nondiabetic retinopathy; DR: diabetic retinopathy; HbA1c: glycated hemoglobin; systolic BP, systolic blood pressure; diastolic BP, diastolic blood pressure; NSAIDs: nonsteroidal anti-inflammatory drugs; eGFR: estimated glomerular filtration rate; CHOL: total cholesterol, TG: triglyceride; HDL-c, high-density lipoprotein cholesterol; LDL-c, low-density lipoprotein cholesterol. All data are presented as the mean ± standard deviation (SD) for normally distributed data or the median (interquartile range) for abnormal distribution. Comparisons between groups were made using a two-tailed t test or Mann-Whitney U test for continuous data and the X^2^ test for categorical data

### Untargeted lipidome-derived biomarkers for diabetic retinopathy: results from the discovery cohort

A total of 1721 lipids were detected. The number of lipids with an RSD (CV) less than or equal to 30% in the QC samples was 1421. The ratio of the number of lipids with CV less than or equal to 30% to the number of all detected lipids in QC samples was 81%.

**Fig. 1:**
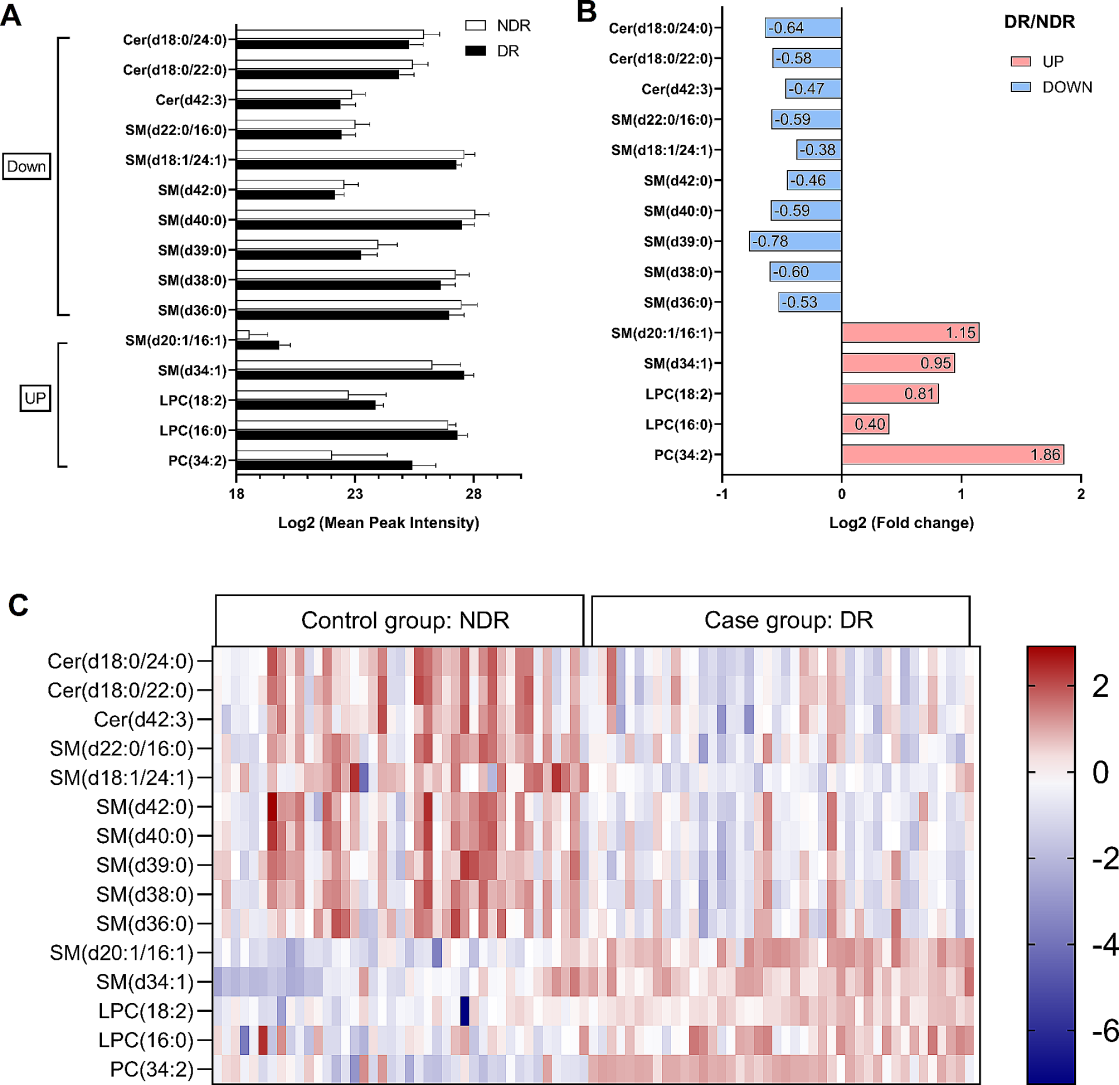
Lipidome-derived markers identified from the discovery cohort. Lipidomic analysis identified fifteen candidate lipids of which serum levels were different between 42 T2DM patients with DR (DR group) and 42 T2DM patients without DR (NDR group) from the discovery cohort. (**A**) Mean peak intensity of lipids was analyzed after Log2 transformation of the data. (**B**) Fold change in DR/NDR was analyzed after Log2 transformation of the data. (**C**) Heatmap showing the distribution of lipid markers. Each row in the figure represents a different lipid, and each column represents a sample. Different colors indicate different intensities, and Log2 conversion was used for the data

Fifteen candidate lipids were identified from the discovery cohort. Compared with those of the NDR group, the levels of three Cer and seven SM were significantly lower in the DR group. In contrast, two SM, two LPC and one PC were significantly higher in the DR group (Fig. 1A and B). More specifically, compared with T2DM patients without DR, T2DM patients with DR showed lower levels of Cer(d18:0/24:0), Cer(d18:0/22:0), Cer(d42:3), SM(d22:0/16:0), SM(d18:1/24:1), SM(d42:0), SM(d40:0), SM(d39:0), SM(d38:0), and SM(d36:0), and higher levels of SM(d20:1/16:1), SM(d34:1), LPC(18:2), LPC(16:0) and PC(34:2). The heat map shows the distribution of these lipids between individuals of the NDR and DR groups (Fig. 1C). The results of ROC analysis and the odds ratios of the lipid markers in the basic logistic regression models are shown in Table 2. The AUC values for the 15 lipids ranged from 0.72 to 0.94. All lipids retained significant ORs after adjusted for CHOL, TG, LDL-c, and HDL-c (adjusted ORs are shown in Table 2). Furthermore, we used ordinal logistic regression, which estimated the odds of being in one higher category of the DR stage (from NDR to PDR) for lipid species, to test the associations between lipid species and DR stage (Table S3; *n* = 42 in the NDR group, *n* = 37 in the NPDR group, *n* = 5 in the PDR group), and we analyzed the data while excluding participants with diabetic macular edema (DME) (*n* = 4 in the DR group), as before, all lipids retained significant ORs (Table S4).

**Table 2:** Results of ROC curve analysis and logistic regression in the discovery cohort

	AUC	*P* value	OR (95% CI)	Adjusted* *P* value	Adjusted* OR (95% CI)
Cer(d18:0/24:0)	0.76	< 0.001	0.20 (0.09–0.47)	< 0.001	0.08 (0.02–0.25)
Cer(d18:0/22:0)	0.74	< 0.001	0.23 (0.10–0.52)	< 0.001	0.10 (0.03–0.30)
Cer(d42:3)	0.72	0.001	0.21 (0.08–0.54)	< 0.001	0.09 (0.02–0.32)
SM(d22:0/16:0)	0.76	< 0.001	0.19 (0.08–0.46)	< 0.001	0.06 (0.01–0.25)
SM(d18:1/24:1)	0.82	< 0.001	0.04 (0.01–0.21)	< 0.001	0.01 (0.00-0.09)
SM(d42:0)	0.73	< 0.001	0.18 (0.07–0.51)	< 0.001	0.05 (0.01–0.20)
SM(d40:0)	0.76	< 0.001	0.15 (0.06–0.39)	< 0.001	0.03 (0.01–0.16)
SM(d39:0)	0.77	< 0.001	0.26 (0.13–0.53)	< 0.001	0.19 (0.08–0.45)
SM(d38:0)	0.78	< 0.001	0.18 (0.08–0.42)	< 0.001	0.05 (0.01–0.19)
SM(d36:0)	0.72	0.001	0.28 (0.13–0.60)	< 0.001	0.19 (0.08–0.48)
SM(d20:1/16:1)	0.94	< 0.001	44.85 (8.86-226.99)	< 0.001	59.91 (9.18-391.17)
SM(d34:1)	0.85	< 0.001	8.39 (3.05–23.14)	< 0.001	7.81 (2.83–21.61)
LPC(18:2)	0.88	< 0.001	59.23 (8.85-396.59)	< 0.001	108.93 (11.07-1072.03)
LPC(16:0)	0.83	< 0.001	26.44 (5.26-132.79)	< 0.001	45.67 (6.79-307.57)
PC(34:2)	0.89	< 0.001	2.76 (1.84–4.15)	< 0.001	3.55 (2.00-6.29)

Cer: ceramides; SM: sphingomyelins; LPC: lysophosphatidylcholines; PC: phosphatidylcholine. The calculation of the area under the curve (AUC) in receiver operating characteristic (ROC) curve analysis is used to evaluate the discriminatory ability of markers. Logistic regression models were applied to assess the relationship between lipid molecules and the presence of diabetic retinopathy. Total cholesterol (CHOL), triglyceride (TG), high density lipoprotein-cholesterol (HDL-c) and low density lipoprotein-cholesterol (LDL-c) were added to multivariate logistic regression to calculate the adjusted* *P* values and odds ratios (ORs).

### Characteristics of the validation cohort and targeted lipidomics analysis

The 15 differential lipids found from the discovery cohort were validated in another set of samples. The clinical characteristics of individuals selected for the validation cohort are shown in Table 3. Most metabolic and clinical features were comparable (Table S5), and there was no significant difference in LDL-c between the DR and NDR groups.

**Table 3:** Clinical characteristics of the validation cohort

	NDR group	DR group	*P* value
Sex (male/total)	63/95	58/95	0.752
Age (years)	57.7 ± 12.4	57.7 ± 12.5	0.878
BMI (kg/m^2^)	24.4 ± 3.6	24.1 ± 4.1	0.854
Diabetes duration (years)	12.9 ± 7.0	12.3 ± 6.4	0.882
HbA1c (%)	8.8 ± 2.1	8.9 ± 2.1	0.775
Hypoglycemic therapy			0.077
Oral hypoglycemic agents	30	18	
Insulin therapy	19	29	
Combination therapy	46	48	
Hypertension status	60/95	59/95	0.881
Antihypertensive agents	56/95	59/95	0.656
Lipid-lowering agents			0.165
Statins	73	80	
Fibrates	7	2	
Others	2	0	
Use of NSAIDs	72/95	72/95	1.000
Systolic BP (mmHg)	136 ± 17	138 ± 18	0.447
Diastolic BP (mmHg)	80 ± 11	81 ± 11	0.774
eGFR (mL/min/1.73 m^2^)	103 ± 15	105 ± 17	0.482
CHOL (mmol/L)	4.00 ± 1.07	3.73 ± 1.04	0.085
TG (mmol/L)	1.30 (1.39)	1.17 (1.14)	0.404
HDL-c (mmol/L)	0.97 ± 0.28	0.95 ± 0.23	0.543
LDL-c (mmol/L)	2.30 ± 0.92	2.18 ± 0.86	0.346

NDR: nondiabetic retinopathy; DR: diabetic retinopathy; HbA1c: glycated hemoglobin; systolic BP, systolic blood pressure; diastolic BP, diastolic blood pressure; NSAIDs: nonsteroidal anti-inflammatory drugs; eGFR: estimated glomerular filtration rate; CHOL: total cholesterol, TG: triglyceride; HDL-c, high-density lipoprotein cholesterol; LDL-c, low-density lipoprotein cholesterol. All data are presented as the mean ± standard deviation (SD) for normally distributed data or the median (interquartile range) for abnormal distribution. Comparisons between groups were made using a two-tailed t test or Mann-Whitney U test for continuous data and the X^2^ test for categorical data

**Fig. 2:**
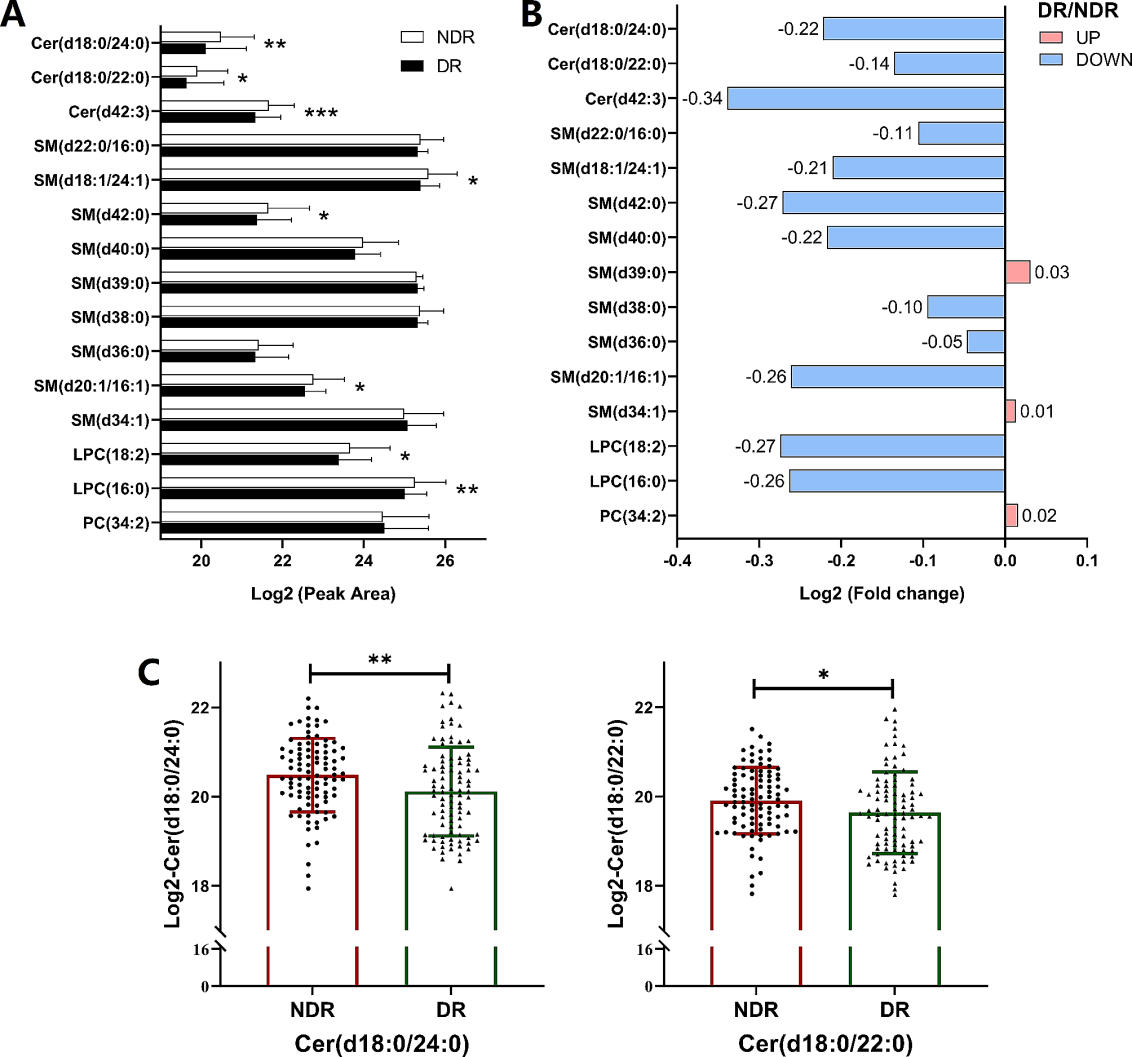
The results of targeted lipidomics analysis in the validation cohort. For the validation cohort, the cases were 95 T2DM patients with DR (DR group), and the control subjects were 95 T2DM patients who had no DR (NDR group). (**A**) Peak area of lipids was analyzed after Log2 transformation of the data. (**B**) Fold change in DR/NDR was analyzed after Log2 transformation of the data. (**C**) The log2 conversion was used for the intensities of Cer(d18:0/24:0) and Cer(d18:0/22:0). All data are presented as the mean ± standard deviation (SD). Each symbol represents an individual participant. *p < 0.05, **p < 0.01, pairwise comparisons of change scores between the groups were evaluated by t test

In the validation cohort, when compared with subjects in the NDR group, T2DM patients with DR showed lower levels of Cer(d18:0/24:0), Cer(d18:0/22:0), Cer(d42:3) and SM(d18:1/24:1) by univariate logistic regression, which was consistent with the results of the discovery cohort. However, the levels of SM(d20:1/16:1), LPC(18:2) and LPC(16:0) were lower in T2DM patients with DR from the validation cohort, opposite to the result obtained in the discovery cohort (Fig. 2A and B). The AUC values for these lipids were higher than 0.61. The other 8 lipids did not significantly differ between the DR and NDR groups in the validation cohort (Table 4). Of note, compared with those in T2DM patients, the peak area (after Log2 transformation) of Cer(d18:0/24:0) (20.48 ± 0.82 vs. 20.12 ± 0.99, *p* = 0.006, Fig. 2C) and Cer(d18:0/22:0) (19.91 ± 0.75 vs. 19.64 ± 0.92, *p* = 0.028, Fig. 2C) remained significantly lower in T2DM patients with DR, and the levels of these two lipids retained significant ORs when adjusted for known risk factors (i.e., CHOL, TG, LDL-c and HDL-c). In the ordinal regression, these two lipids maintained significant ORs (Table S7, *n* = 95 in the NDR group, *n* = 87 in the NPDR group; *n* = 8 in the PDR group), and were also significant while excluding patients with DME (Table S6, *n* = 2 in the DR group). These findings imply that levels of Cer(d18:0/24:0) and Cer(d18:0/22:0) were independent markers for T2DM patients with DR in both the discovery cohort (Table 2) and validation cohort (Table 4).

**Table 4:** Results of ROC curve analysis and logistic regression in the validation cohort

	AUC	*P* value	OR (95% CI)	Adjusted* *P* value	Adjusted* OR (95% CI)
Cer(d18:0/24:0)	0.62	**0.007**	0.64 (0.46–0.89)	**0.002**	0.45 (0.27–0.75)
Cer(d18:0/22:0)	0.61	**0.030**	0.68 (0.48–0.96)	**0.022**	0.65 (0.45–0.94)
Cer(d42:3)	0.65	**0.001**	0.42 (0.25–0.68)	**0.006**	0.62 (0.44–0.87)
SM(d22:0/16:0)	0.64	0.293	0.61 (0.24–1.53)	0.340	0.66 (0.28–1.55)
SM(d18:1/24:1)	0.65	**0.038**	0.51 (0.27–0.96)	0.066	0.54 (0.28–1.04)
SM(d42:0)	0.62	0.050	0.72 (0.51-1.00)	0.059	0.72 (0.51–1.01)
SM(d40:0)	0.62	0.080	0.67 (0.43–1.05)	0.125	0.70 (0.45–1.10)
SM(d39:0)	0.57	0.151	4.20 (0.59–29.80)	0.112	5.16 (0.68–38.87)
SM(d38:0)	0.63	0.395	0.72 (0.34–1.54)	0.465	0.77 (0.37–1.57)
SM(d36:0)	0.55	0.483	0.88 (0.62–1.25)	0.574	0.90 (0.63–1.29)
SM(d20:1/16:1)	0.63	**0.036**	0.57 (0.34–0.96)	**0.042**	0.56 (0.32–0.98)
SM(d34:1)	0.57	0.461	1.14 (0.81–1.61)	0.633	1.10 (0.75–1.59)
LPC(18:2)	0.63	**0.044**	0.70 (0.49–0.99)	**0.046**	0.68 (0.47–0.99)
LPC(16:0)	0.67	**0.009**	0.47 (0.26–0.83)	**0.017**	0.48 (0.27–0.88)
PC(34:2)	0.51	0.760	1.04 (0.80–1.35)	0.882	1.02 (0.78–1.34)

Cer: ceramides; SM: sphingomyelins; LPC: lysophosphatidylcholines; PC: phosphatidylcholine. The calculation of the area under the curve (AUC) in receiver operating characteristic (ROC) curve analysis is used to evaluate the discriminatory ability of markers. Logistic regression models were applied to assess the relationship between lipid molecules and the presence of diabetic retinopathy. Total cholesterol (CHOL), triglyceride (TG), high density lipoprotein-cholesterol (HDL-c) and low density lipoprotein-cholesterol (LDL-c) were added to multivariate logistic regression to calculate the adjusted* *P* values and odds ratios (OR).

## Discussion

DR is the most common microvascular complication of diabetes and the main factor contributing to visual impairment in working-age individuals [3]. T2DM patients often develop DR despite of proper control of systemic risk factors, indicating the involvement of other pathogenic factors for DR development. To find new and more effective strategies for preventing and treating DR, it is necessary for us to identify novel biomarkers for DR screening or detection. Lipidomics will aid in understanding the mechanism of DR at various stages of the disease, early diagnosis, and the identification of new therapeutic targets. In this study, by using two clinical cohorts, we found that the serum lipidomic profiles in T2DM patients with DR showed significant differences from those in T2DM patients without DR. The differential lipid species in the DR group were linked to disturbances in sphingolipid metabolism. Compared with those in the NDR group, the levels of Cer(d18:0/24:0) and Cer(d18:0/22:0) were significantly lower in the DR group after adjusting for covariates, i.e. known risk factors in both the discovery and validation cohorts. These findings suggest that these two lipid species may be potential serological markers for the diagnosis of DR in patients with T2DM.

In this study, we found two ceramide molecules that were significantly lower in T2DM patients with DR, indicating that they may have disturbed ceramide metabolism compared to T2DM patients without DR. Ceramide is sphingolipid [11] and can be found in VLDL, LDL, and HDL. Consistent with our findings, Fort et al. found a significantly lower abundance of Cer in central retinal tissue obtained postmortem from T2DM patients with DR compared to those without DR [26]. Similarly, ceramide levels were shown to be lower and glucosylceramide levels higher in the retinas of diabetic rodents [27]. This indicates that diabetes reduces the retinal ceramide content and may suggest that dysregulated sphingolipid metabolism may cause retinal resistance to insulin action [27]. These findings imply that ceramide is diverted from the overall pools of retinal sphingolipids toward the glycosylated forms due to hyperglycemia. In contrast, Levitsky et al. found that diabetes-induced increases in mitochondrial ceramide led to impaired mitochondrial function in the retinal pigment epithelial (RPE) cells of the retina [28], and disruption of the blood-retinal barrier might be caused by diabetes-induced overexpression of acid sphingomyelinase. Additionally, inflammation is a common underlying factor in DR, and inflammation generates Cer from SM in the serum membrane. This induces death receptor ligand formation and leads to apoptosis of RPE and photoreceptor cells [29]. In addition to diabetes, circulating Cer was shown to strongly correlate with future adverse cardiovascular events. It has recently been discovered that in individuals with atherosclerotic CVD, serum levels of specific Cer species can predict the future risk of cardiovascular death. In the Corogene study, higher concentrations of Cer(d18:1/16:0), Cer(d18:1/18:0), and Cer(d18:1/24:1) and lower concentrations of Cer(d18:1/24:0) were associated with a higher risk of fatal myocardial infarction [30]. Our study found that Cer(d18:0/24:0) and Cer(d18:0/22:0) were significantly lower in T2DM patients with DR compared to those without DR, which suggests that different numbers of carbons and double bonds in ceramides might play differential roles in DR and CVD. The distinct ceramides and ceramide metabolites involved in metabolic regulation play unanticipated roles [31]. Watt et al. discovered that circulating ceramides present in LDL particles were sufficient to induce insulin resistance in vitro and in vivo [32]. However, how these two identified ceramides influence lipid metabolism in T2DM remains unclear and needs further exploration. Thus, disturbed Cer metabolism may contribute to dysfunction in DR, and therapeutic strategies to restore normal Cer metabolism might be an effective approach for treatment of DR.

In the discovery cohort, LPC(18:2) and LPC(16:0) were significantly higher in T2DM patients with DR. However, these two lipids were significantly lower in DR in the validation cohort. The previous findings point to a change in sphingolipid composition between control and T2DM [33]. LPC is an inflammatory phospholipid and an important atherogenic substance in LDL that contributes to diabetic complications [34]. Lipoprotein-associated phospholipase A2 (Lp-PLA2) plays a crucial role in diabetes-related retinal vasopermeability, a response mediated by LPC, and inhibiting Lp-PLA2 reduces diabetes-induced retinal vasopermeability [35]. LPC O-16:0, LPC P-16:0, LPC O-18:0, and LPC 18:1 were all found to be inversely related to incident T2DM [36]. The differences between the discovery and validation cohorts may be related to the populations studied, medications used, and stages of diabetic retinopathy [37].

There are some limitations of this study. First, only a Chinese ethnic group was selected, and future validation of our findings in other races or ethnic groups is warranted. Second, instead of chronic risk factors associated with the development of DR, some of the identified lipid markers might only represent temporary metabolic perturbations in this cross-sectional study. Third, the exact mechanism of DR development in patients with T2DM through which ceramide functions has not been explained. Therefore, more extensive preclinical and clinical studies are needed to clarify the mechanisms behind the potential effects of specific lipids.

Overall, the deregulation of sphingolipid metabolism in the diabetic retina appears to be a significant and seldom-studied element of DR pathophysiology. The precise mechanism underlying this disease is still unknown and requires further investigation. We showed the potential value of lipidomics research in understanding the pathophysiology of DR, and the results suggest that lipidomics profiling may be capable of identifying early-stage DR diagnostic indicators in high-risk Chinese populations. In addition, the findings from this study may help in the elucidation of new therapeutic targets for DR prevention and treatment.

### Electronic supplementary material

Below is the link to the electronic supplementary material.

Supplementary Material 1

## Data Availability

All relevant data and materials have been included in the article and its supplementary data files. Further inquiries can be directed to the corresponding authors.

## Abbreviations

T2DM: Type 2 diabetes mellitus
DR: Diabetic retinopathy
LC–MS/MS: Liquid chromatography–mass spectrometry
PLS-DA: Partial least squares discriminant analysis
VIP: Variable importance in the projection
PSM: Propensity score matching
Cer: Ceramides
SM: Sphingomyelins
PC: Phosphatidylcholine
LPC: Lysophosphatidylcholines
MRM: Multiple reaction monitoring
SBP: Systolic blood pressure
DBP: Diastolic blood pressure
CHOL: Total cholesterol
TG: Triglyceride
HDL-c: High density lipoprotein-cholesterol
LDL-c: Low density lipoprotein-cholesterol
UA: Uric acid
AST: Aspartate aminotransferase
ALT: Alanine aminotransferase
ALP: Alkaline phosphatase
GGT: Gamma-glutamyl transpeptidase
TBIL: Total bilirubin
DBIL: Direct bilirubin
TP: Total protein
ALB: Albumin
GLU: Glucose
BUN: Blood urea nitrogen
CRE: Creatinine
