# Supplementary material for: Lipidomic studies revealing serological markers associated with the occurrence of retinopathy in type 2 diabetes
Source: J Transl Med. 2024 May 13;22:448. doi: 10.1186/s12967-024-05274-9 (PMC11089707; doi:10.1186/s12967-024-05274-9)
Supplement: Supplementary file 1 — Supplementary Material 1 [file 12967_2024_5274_MOESM1_ESM.docx]

**Supplemental materials**

**Table S1 Detailed MRM conditions and retention times of targeted lipids**

| Name | Precursor m/z | Product m/z | Retention  Time (min) | Ion  mode | DP (eV) | CE  (eV) |
| --- | --- | --- | --- | --- | --- | --- |
| Cer(d18:0/24:0) | 652.66 | 284.29 | 6.79 | Positive | 100 | 35 |
| Cer(d18:0/22:0) | 624.63 | 284.29 | 7.05 | Positive | 100 | 35 |
| Cer(d42:3) | 646.61 | 628.61 | 6.43 | Positive | 100 | 18 |
| SM(d22:0/16:0) | 761.65 | 184.21 | 5.57 | Positive | 100 | 28 |
| SM(d18:1/24:1) | 813.68 | 184.21 | 6.14 | Positive | 100 | 28 |
| SM(d42:0) | 817.72 | 184.21 | 6.45 | Positive | 100 | 28 |
| SM(d40:0) | 789.69 | 184.21 | 5.88 | Positive | 100 | 28 |
| SM(d39:0) | 755.69 | 184.21 | 5.43 | Positive | 100 | 28 |
| SM(d38:0) | 761.65 | 184.21 | 5.57 | Positive | 100 | 28 |
| SM(d36:0) | 733.62 | 184.21 | 5.31 | Positive | 100 | 28 |
| SM(d20:1/16:1) | 729.58 | 184.21 | 5.33 | Positive | 100 | 28 |
| SM(d34:1) | 703.58 | 184.21 | 5.31 | Positive | 100 | 28 |
| LPC(18:2) | 520.34 | 184.21 | 1.22 | Positive | 100 | 30 |
| LPC(16:0) | 495.34 | 184.21 | 1.47 | Positive | 100 | 30 |
| PC(34:2) | 758.57 | 184.21 | 5.43 | Positive | 100 | 32 |
| Cer(d18:1/16:0)-d7 | 545.91 | 284.29 | 5.81 | Positive | 100 | 28 |
| SM(d18:1/18:1)­-d9 | 738.71 | 184.21 | 5.31 | Positive | 100 | 28 |
| LPC(18:1)-d7 | 529.41 | 184.21 | 1.52 | Positive | 100 | 30 |
| PC(15:0/18:1)-d7 | 753.61 | 184.21 | 5.43 | Positive | 100 | 32 |

Cer: ceramide; SM: sphingomyelin; LPC: lysophosphatidylcholine; PC: phosphatidylcholine; DP: declustering potentials; CE: collision energy.

**Table S2 Clinical characteristics of the discovery cohort**

|  | NDR group | DR group | P value |
| --- | --- | --- | --- |
| AST (U/L) | 21.00 (14.50) | 17.50 (8.30) | **0.014** |
| ALT (U/L) | 22.00 (19.50) | 18.50 (13.30) | 0.057 |
| ALP (U/L) | 67.79±24.86 | 68.12±22.62 | 0.949 |
| GGT (U/L) | 25.00 (19.00) | 22.50 (17.30) | 0.460 |
| TBIL(µmol/L) | 12.71±6.14 | 10.51±3.38 | **0.046** |
| DBIL (µmol/L) | 3.93±2.59 | 3.11±1.47 | 0.082 |
| TP (g/L) | 66.58±5.61 | 65.72±5.84 | 0.493 |
| ALB (g/L) | 40.63±3.37 | 40.31±4.13 | 0.695 |
| GLU (mmol/L) | 7.55±2.69 | 7.59±2.92 | 0.944 |
| UA (µmol/L) | 318.38±85.44 | 332.29±75.19 | 0.431 |
| BUN (mmol/L) | 5.47±1.43 | 6.61±1.74 | **0.004** |
| CRE (µmol/L) | 58.07±17.01 | 64.14±18.87 | 0.125 |

NDR: Non-diabetic retinopathy; DR: diabetic retinopathy; AST: aspartate aminotransferase; ALT: alanine aminotransferase; ALP: alkaline phosphatase; GGT: gamma-glutamyl transpeptidase; TBIL: total bilirubin; DBIL: direct bilirubin; TP: total protein; ALB: albumin; GLU: glucose; UA: uric acid; BUN: blood urea nitrogen; CRE: creatinine. All data are presented as the mean ± standard deviation (SD) for normal distribution or the median (interquartile range) for abnormal distribution. Comparisons between groups were made using a two-tailed t test or Mann-Whitney U test.

**Table S3 Results of ordinal logistic regression in the discovery cohort**

|  | P value | OR (95% CI) |
| --- | --- | --- |
| Cer(d18:0/24:0) | <0.001 | 0.20(0.09-0.44) |
| Cer(d18:0/22:0) | <0.001 | 0.24(0.11-0.51) |
| Cer(d42:3) | <0.001 | 0.20(0.09-0.45) |
| SM(d22:0/16:0) | <0.001 | 0.16(0.07-0.36) |
| SM(d18:1/24:1) | <0.001 | 0.03 (0.01-0.17) |
| SM(d42:0) | <0.001 | 0.14(0.05-0.40) |
| SM(d40:0) | <0.001 | 0.12(0.05-0.32) |
| SM(d39:0) | <0.001 | 0.25(0.13-0.48) |
| SM(d38:0) | <0.001 | 0.15(0.07-0.35) |
| SM(d36:0) | <0.001 | 0.22 (0.10-0.47) |
| SM(d20:1/16:1) | <0.001 | 14.85(5.18-42.57) |
| SM(d34:1) | <0.001 | 5.70(2.59-12.52) |
| LPC(18:2) | <0.001 | 36.83(8.20-165.47) |
| LPC(16:0) | <0.001 | 20.36(4.71-88.02) |
| PC(34:2) | <0.001 | 2.72(1.83-4.04) |

Cer: ceramide; SM: sphingomyelin; LPC: lysophosphatidylcholine; PC: phosphatidylcholine. The area under the receiver operating characteristic (ROC) curve (AUC) was calculated to evaluate the discriminatory ability of the markers. Ordinal logistic regression models were used to assess the relationships between lipid molecules and DR stages (NDR, NPDR and PDR).

**Table S4 Results of ROC curve analysis and logistic regression in the discovery cohort excluding patients with DME**

|  | AUC | P value | OR (95% CI) |
| --- | --- | --- | --- |
| Cer(d18:0/24:0) | 0.76 | <0.001 | 0.20 (0.07-0.48) |
| Cer(d18:0/22:0) | 0.74 | <0.001 | 0.21 (0.09-0.51) |
| Cer(d42:3) | 0.71 | 0.002 | 0.21 (0.0800.57) |
| SM(d22:0/16:0) | 0.74 | 0.001 | 0.22 (0.09-0.52) |
| SM(d18:1/24:1) | 0.81 | 0.001 | 0.04 (0.01-0.25) |
| SM(d42:0) | 0.71 | 0.003 | 0.21 (0.07-0.60) |
| SM(d40:0) | 0.74 | <0.001 | 0.17 (0.06-0.45) |
| SM(d39:0) | 0.75 | 0.001 | 0.28 (0.14-0.58) |
| SM(d38:0) | 0.75 | <0.001 | 0.20 (0.08-0.48) |
| SM(d36:0) | 0.72 | 0.004 | 0.32 (0.15-0.70) |
| SM(d20:1/16:1) | 0.51 | <0.001 | 74.35 (10.60-521.38) |
| SM(d34:1) | 0.85 | <0.001 | 8.04 (2.93-22.06) |
| LPC(18:2) | 0.88 | <0.001 | 52.00 (7.85-344.35) |
| LPC(16:0) | 0.84 | <0.001 | 24.60 (4.90-123.62) |
| PC(34:2) | 0.89 | <0.001 | 2.67 (1.78-4.02) |

DME: diabetic macular edema; Cer: ceramide; SM: sphingomyelin; LPC: lysophosphatidylcholine; PC: phosphatidylcholine. The area under the receiver operating characteristic (ROC) curve (AUC) was calculated to evaluate the discriminatory ability of the markers. Logistic regression models were used to assess the associations between lipid molecules and the presence of diabetic retinopathy.

**Table S5 Clinical characteristics of the validation cohort**

|  | NDR group | DR group | P value |
| --- | --- | --- | --- |
| AST (U/L) | 22.00 (9.00) | 18.00 (7.00) | 0.527 |
| ALT (U/L) | 20.00 (18.00) | 19.00 (12.00) | 0.223 |
| ALP (U/L) | 69.66±29.42 | 77.78±34.32 | 0.090 |
| GGT (U/L) | 22.00 (26.00) | 18.50 (14.00) | 0.081 |
| TBIL(µmol/L) | 3.25±1.64 | 3.60±2.59 | 0.282 |
| DBIL (µmol/L) | 8.62±3.47 | 8.32±3.29 | 0.557 |
| TP (g/L) | 64.21±4.62 | 63.05±5.15 | 0.111 |
| ALB (g/L) | 38.82±4.03 | 39.30±8.91 | 0.633 |
| GLU (mmol/L) | 7.88±3.73 | 8.90±4.74 | 0.109 |
| UA (µmol/L) | 329.11±96.89 | 316.77±95.01 | 0.384 |
| BUN (mmol/L) | 5.71±1.94 | 6.14±1.90 | 0.135 |
| CRE (µmol/L) | 59.68±16.34 | 56.98±16.69 | 0.270 |

NDR: Non-diabetic retinopathy; DR: diabetic retinopathy; AST: aspartate aminotransferase; ALT: alanine aminotransferase; ALP: alkaline phosphatase; GGT: gamma-glutamyl transpeptidase; TBIL: total bilirubin; DBIL: direct bilirubin; TP: total protein; ALB: albumin; GLU: glucose; UA: uric acid; BUN: blood urea nitrogen; CRE: creatinine. All data are presented as the mean ± standard deviation (SD) for normally distributed data or the median (interquartile range) for abnormal distribution. Comparisons between groups were made using a two-tailed t test or Mann-Whitney U test.

**Table S6 Results of ROC curve analysis and logistic regression in the validation cohort excluding patients with DME**

|  | AUC | P value | OR (95% CI) |
| --- | --- | --- | --- |
| Cer(d18:0/24:0) | 0.62 | 0.008 | 0.65(0.47-0.89) |
| Cer(d18:0/22:0) | 0.61 | 0.032 | 0.68(0.48-0.97) |
| Cer(d42:3) | 0.65 | 0.001 | 0.43(0.26-0.70) |
| SM(d22:0/16:0) | 0.62 | 0.313 | 0.63(0.26-1.54) |
| SM(d18:1/24:1) | 0.65 | 0.046 | 0.52 (0.27-0.99) |
| SM(d42:0) | 0.62 | 0.048 | 0.72(0.51-1.00) |
| SM(d40:0) | 0.61 | 0.095 | 0.69(0.44-1.07) |
| SM(d39:0) | 0.43 | 0.143 | 4.34(0.61-30.86) |
| SM(d38:0) | 0.62 | 0.418 | 0.74(0.35-1.55) |
| SM(d36:0) | 0.54 | 0.511 | 0.89(0.63-1.26) |
| SM(d20:1/16:1) | 0.62 | 0.039 | 0.57(0.34-0.97) |
| SM(d34:1) | 0.57 | 0.479 | 1.13(0.80-1.60) |
| LPC(18:2) | 0.62 | 0.047 | 0.70(0.49-1.00) |
| LPC(16:0) | 0.67 | 0.010 | 0.47(0.26-0.83) |
| PC(34:2) | 0.50 | 0.753 | 1.04(0.80-1.35) |

DME: diabetic macular edema; Cer: ceramide; SM: sphingomyelin; LPC: lysophosphatidylcholine; PC: phosphatidylcholine. The area under the receiver operating characteristic (ROC) curve (AUC) was calculated to evaluate the discriminatory ability of the markers. Logistic regression models were used to assess the associations between lipid molecules and the presence of diabetic retinopathy.

**Table S7 Results of ordinal logistic regression in the validation cohort**

|  | P value | OR (95% CI) |
| --- | --- | --- |
| Cer(d18:0/24:0) | 0.015 | 0.68(0.50-0.93) |
| Cer(d18:0/22:0) | 0.050 | 0.72 (0.51-0.99) |
| Cer(d42:3) | <0.001 | 0.39(0.24-0.64) |
| SM(d22:0/16:0) | 0.349 | 0.68(0.30-1.53) |
| SM(d18:1/24:1) | 0.052 | 0.55 (0.30-1.01) |
| SM(d42:0) | 0.093 | 0.76(0.55-1.05) |
| SM(d40:0) | 0.123 | 0.72(0.48-1.09) |
| SM(d39:0) | 0.213 | 3.30(0.50-21.61) |
| SM(d38:0) | 0.467 | 0.77(0.38-1.56) |
| SM(d36:0) | 0.720 | 0.94(0.67-1.32) |
| SM(d20:1/16:1) | 0.055 | 0.61(0.37-1.01) |
| SM(d34:1) | 0.440 | 1.14(0.81-1.61) |
| LPC(18:2) | 0.052 | 0.72(0.51-1.00) |
| LPC(16:0) | 0.017 | 0.52(0.31-0.89) |
| PC(34:2) | 0.632 | 1.06(0.83-1.37) |

Cer: ceramide; SM: sphingomyelin; LPC: lysophosphatidylcholine; PC: phosphatidylcholine. The area under the receiver operating characteristic (ROC) curve (AUC) was calculated to evaluate the discriminatory ability of the markers. Ordinal logistic regression models were used to assess the relationships between lipid molecules and DR stages (NDR, NPDR and PDR).
